# Expanding diagnostic approach in patients with osteonecrosis

**DOI:** 10.1186/s13075-021-02533-8

**Published:** 2021-05-25

**Authors:** José A. Gómez-Puerta, Pilar Peris

**Affiliations:** Rheumatology Department, Hospital Clinic de Barcelona, Villarroel 170, 08036 Barcelona, Spain

To the editor,

We read with interest the manuscript recently published by Yokota and colleagues [1]. Whole-body MRI (WB-MRI) is an attractive tool for the diagnosis of patients with high risk of developing multifocal osteonecrosis (ON). In many cases, concordance between symptoms and diagnostic images is poor; therefore, as suggested by the authors, high suspicion is needed. Compared with whole-body bone scintigraphy (WB-BS), in this study, a significant proportion of patients with asymptomatic ON were identified using WB-MRI (being the knees and the shoulders the most frequent underdiagnosed affected locations), thereby indicating that this method should be used for a more correct diagnostic and therapeutic approach.

Treatment of patients with ON, especially those with multifocal ON, remains a great challenge for clinicians. Although the authors indicated the need to better identify the patients at risk for this extensive type of ON, they did not mention any additional study related to the presence of associated coagulation abnormalities, which are a relatively common finding as described previously by our group and others in patients with multifocal ON [2, 3]. Indeed, in a previous observational study in a series of 29 patients with multifocal ON [2], we observed the presence of coagulation abnormalities in nearly 50% of the patients after performing a wide thrombophilic profile panel. Of note, most of the patients (65%) also had a present or previous history of glucocorticoid treatment indicating the importance of both glucorticoid treatment and coagulation abnormalities as associated risk factors. In our study, the most common prothrombotic alterations were high factor VIII levels and the presence of antiphospholipid antibody.

Also, of interest in relation to the Yokota study is the influence of the WB-MRI diagnosis on the clinical approach to these patients. Did the patients diagnosed with multifocal ON in the present study receive additional or differential treatment compared with those with oligarticular forms of ON—particularly when asymptomatic?

The present study provides a useful tool for diagnosing this clinical entity, which seems to be more frequent than previously indicated, with the use of a more sensitive test such as WB-MRI. Nevertheless, the identification of high-risk patients for this complication is essential. Therefore, in addition to the wider and more accurate and sensitive WB-MRI imaging approach, complementary tests of pro-thrombotic factors are recommended to better identify patients at high risk of multifocal ON who could be candidates for antiplatelet, antiaggregant, or even anticoagulant therapy, among others [4, 5].

## Data Availability

Data from original article cited is available under request.
